# Supplementary material for: Mapping of endosomal proximity proteomes reveals Retromer as a hub for RAB GTPase regulation
Source: Nat Commun. 2025 Jul 30;16:6990. doi: 10.1038/s41467-025-61802-1 (PMC12311110; doi:10.1038/s41467-025-61802-1)
Supplement: Supplementary file 1 — Supplementary Information [file 41467_2025_61802_MOESM1_ESM.pdf]

Supplementary Figure 1

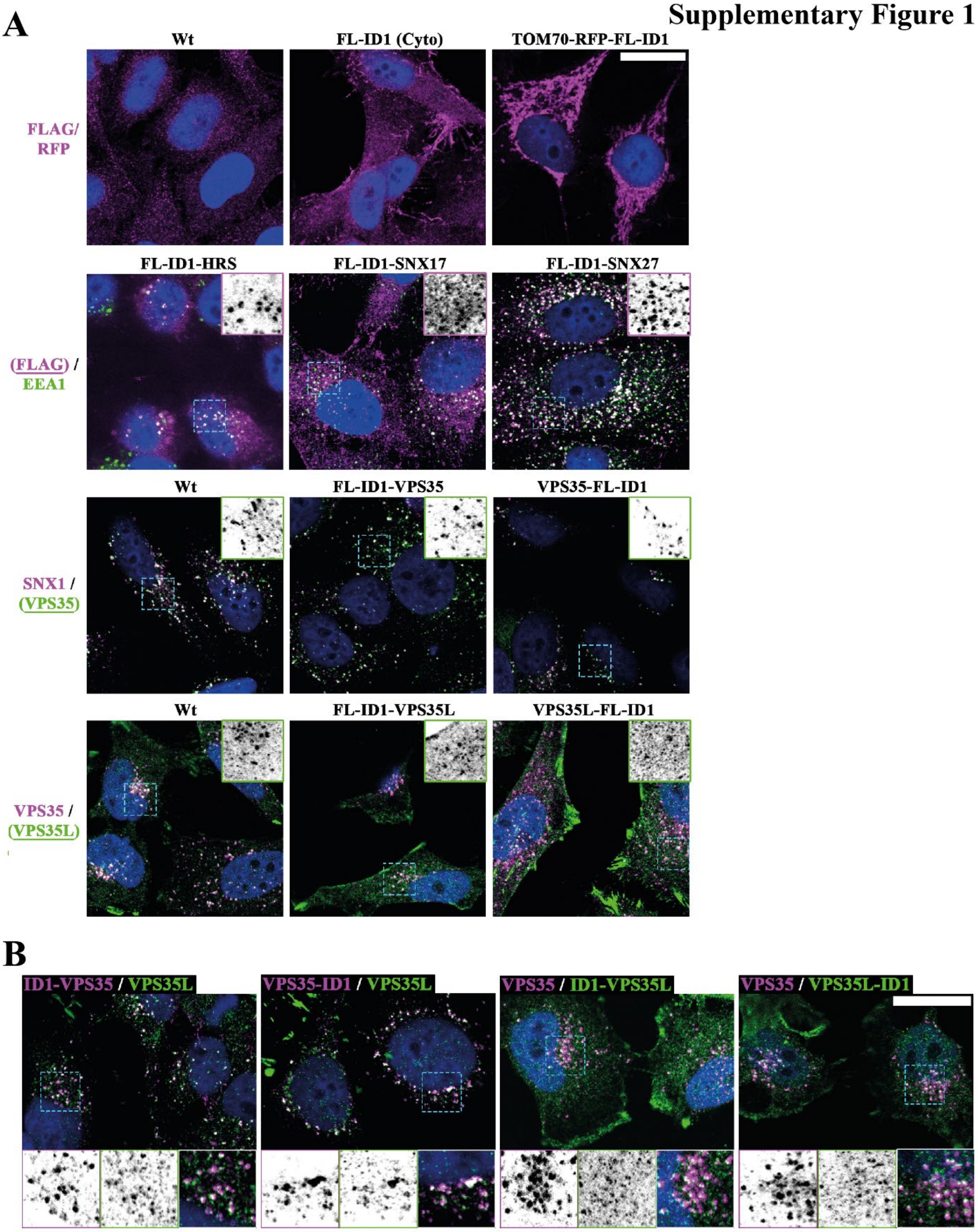

Supplementary Figure 1. (A) Confocal images showing validation of localization of BioID1-tagged proteins/locations using specific antibodies or anti-FLAG. The FLAG epitope is coded just before the BioID1 sequence and 'FL-ID1' is abbreviation for FLAG epitope followed by BioID1 biotin ligase. **(B)** Confocal images showing colocalization between Retromer (VPS35) and Retriever (VPS35L) complexes in cells expressing the different BioID1-tagged versions of VPS35 or VPS35L. Scale bar - 20 μm.

Supplementary Figure 2

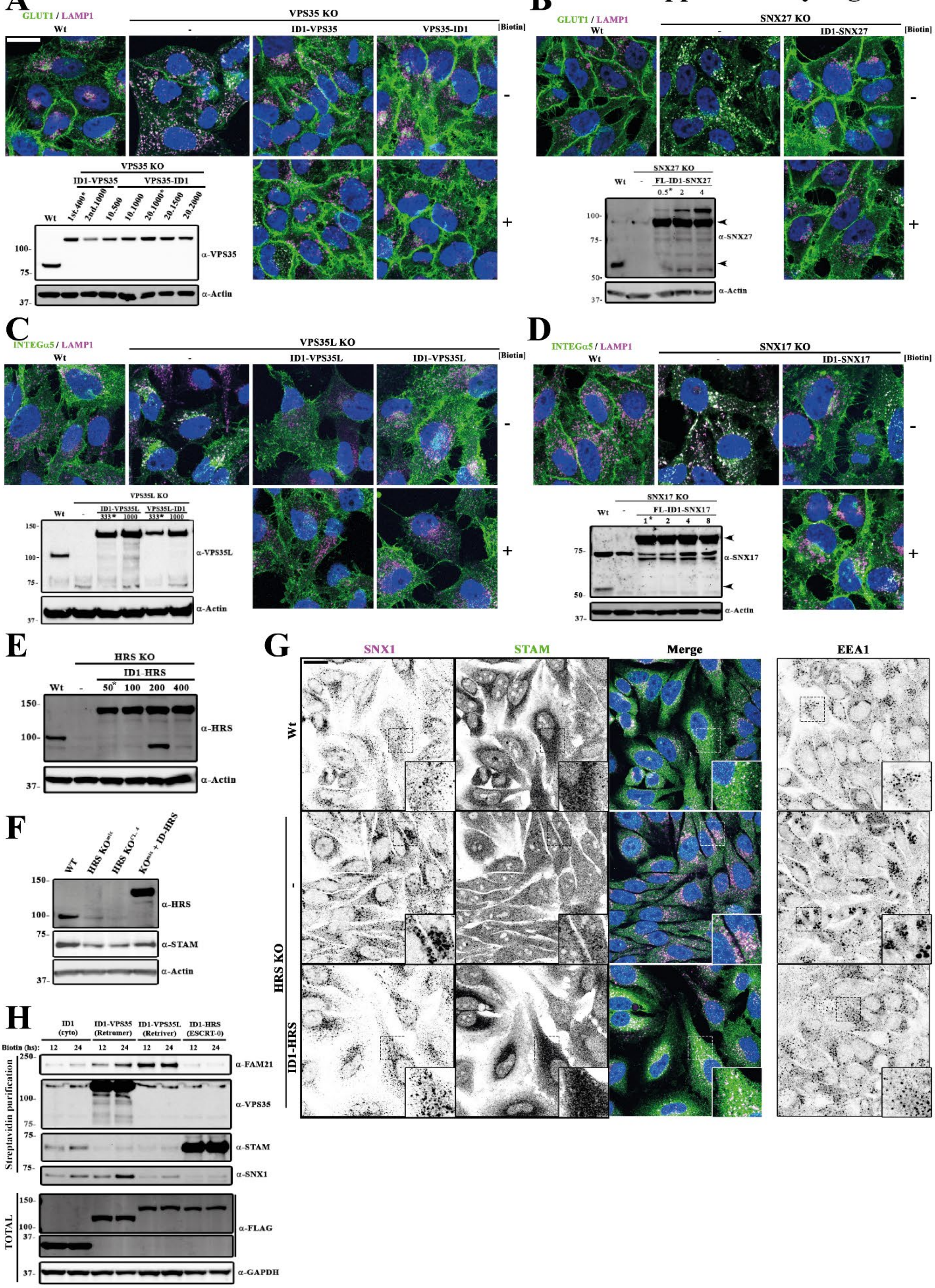

Supplementary Figure 2. (A) Validation of BioID1 Retromer (VPS35) engineered cells lines; ID1-VPS35 and VPS35-ID1. Previously characterized HeLa VPS35 KO cell line was transduced with lentivirus to express ID1-VPS35 or VPS35-ID1. By means of viral titration and puromycin selection populations with VPS35 levels comparable to the endogenous were selected. In the left bottom western blot (WB), asterisk indicates cell populations used for later studies. The functionality of the chimeras ID1-VPS35 and VPS35-ID1 was confirmed by analyzing the rescue of GLUT1 lysosomal missorting observed in HeLa VPS35 KO cells. The confocal images show colocalization of GLUT1 with lysosomal marker LAMP1. GLUT1 recovers its plasma membrane localization when VPS35 BioID1 versions are expressed, at basal conditions (top row images) and after 24 hrs incubation with 50 mM biotin (bottom row images). **(B)** Validation of ID1-SNX27 engineered cell line. Previously characterized HeLa SNX27 KO cell line was transduced with lentivirus to express ID1-SNX27 and by means of viral titration and puromycin selection we isolated population with SNX2 7 levels comparable to the endogenous. In the left bottom WB, asterisk indicated cell population used for later studies. The functionality of the chimera ID1-SNX27 was confirmed by analyzing the rescue of GLUT1 lysosomal missorting observed in HeLa SNX27 KO cells, following the same procedure as in (A). **(C)** Validation of BioID1 Retriever (VPS35L) engineered cells lines; ID1-VPS35L and VPS35L-ID1. Previously characterized HeLa VPS35L KO cell line was transduced with lentivirus to express ID1-VPS35L or VPS35L-ID1 and by means of viral titration and puromycin selection we isolated populations with levels comparable to the endogenous VPS35L (left bottom WB, asterisk indicated cell populations used for later studies). The functionality of the chimeras ID1-VPS35L and VPS35L-ID1 was confirmed by analyzing the rescue of α5-integrin lysosomal missorting observed in HeLa VPS35L KO cells. The confocal images show colocalization of α5-integrin with lysosomal marker LAMP1. α5-integrin recovers its plasma membrane localization when VPS35L BioID1 versions are expressed, at basal conditions (top row images) and after 24 hrs incubation with 50 mM biotin (bottom row images). **(D)** Validation of ID1-SNX17 engineered cell line. Previously characterized HeLa SNX17 KO cell line was transduced with lentivirus to express ID1-SNX17 and by means of viral titration and puromycin selection we isolated populations with the closest possible levels compared to endogenous SNX17 (left bottom WB, asterisk indicated cell population used for later studies). The functionality of the chimera ID1-SNX17 was confirmed by analyzing the rescue of α5-integrin lysosomal missorting observed in HeLa SNX17 KO cells, following the same procedure as in (C). **(E-G)** Validation of ID1-HRS engineered cell line. For the generation of HeLa CRISPR KO for HRS/HGS, we use a guide against HRS/HGS exon 3 (Table S1). **(E)** Single clonal HRS KO cell line was transduced with lentivirus to express ID1-HRS and by means of viral titration and puromycin selection we isolated populations with the closest possible levels compared to endogenous HRS/HGS; asterisk indicated cell population used for later studies. **(F)** Western blot showing the rescue of total levels of STAM1/2 protein when ID1-HRS version is expressed. HRS/HGS and STAM1/2 form the ESCRT-0 complex. **(G)** Confocal images showing the rescue of SNX1 endosome morphology, STAM1/2 localization (left panels) and morphology of early endosomal marker EEA1 (right panels) when ID1-HRS version is expressed. **(H)** Western blot showing comparison of the biotinylated proteins after 12- or 24-hours incubation with 50 mM biotin, lysis and streptavidin purification among different

cell lines. Top part shows biotinylated proteins, and bottom part shows total protein levels. Scale bar - 20 μm.

Supplementary Figure 3

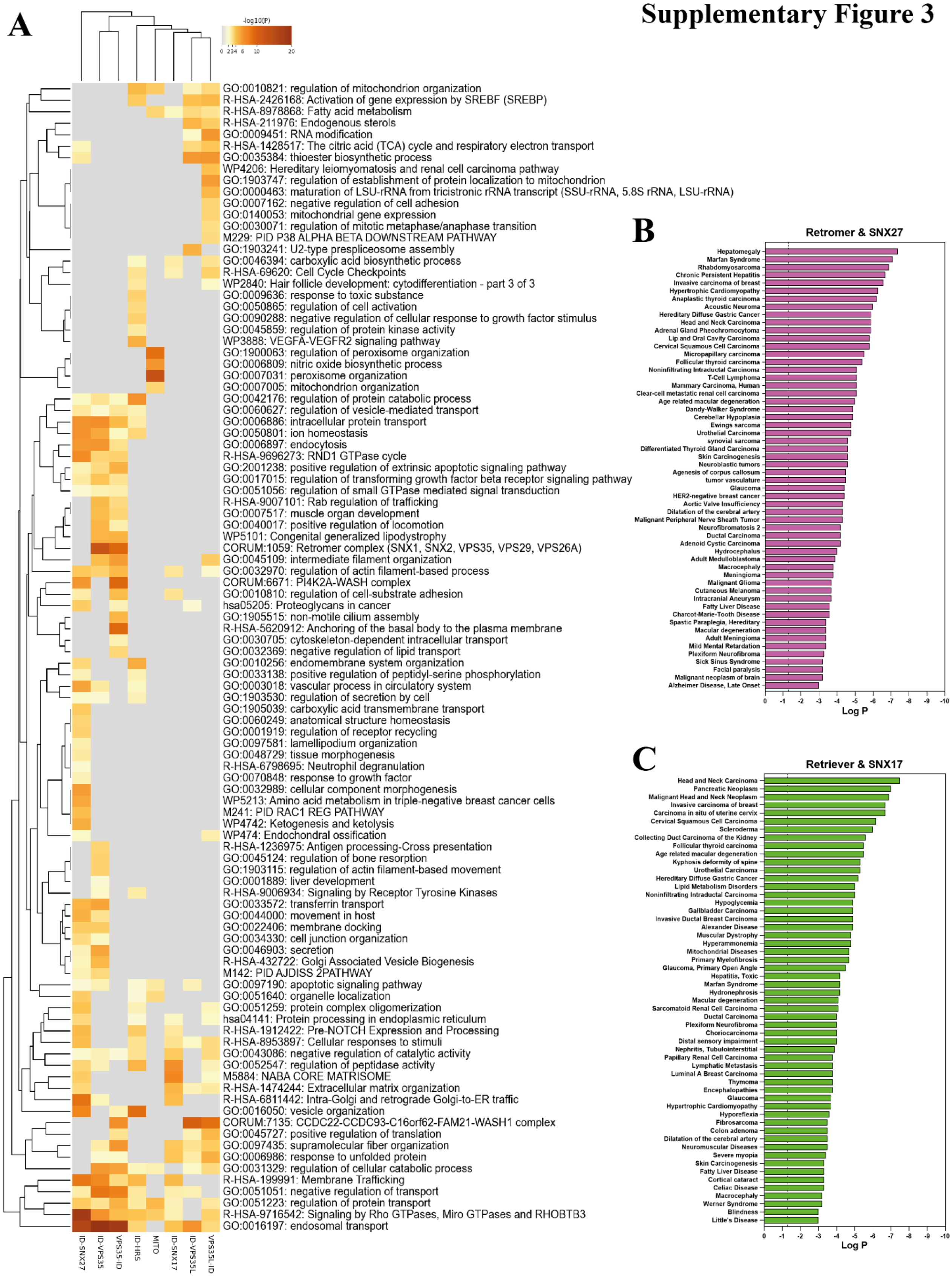

Supplementary Figure 3. Gene ontology (GO) comparison among all proximity proteomes; extended versions. (A) Heat map representing the comparison of the main GO terms associated to

each proximity proteome. Significant proteins (p-value < 0.05) with FC ≥ 0.8 detected in proximity to every BioID1 when compared with cytosolic BioID1 were analyzed with Metascape to represent pathway enrichment, using a hypergeometric test and Benjamini-Hochberg correction, pathway enrichment p-value is represented in -Log10 scale. The colour gradient represents the enrichment significance for each GO term ($-Log_{10}$); for reference 1.3 $-Log_{10}$ is equal to 0.05 p-value. **(B-C)** Retromer-SNX27 (B) and Retriever-SNX17 (C) proximity proteomes show enrichment for multiple disease associated GO terms from DisGeneNET. Significant proteins (p-value < 0.05) with FC ≥ 0.8 detected in proximity to SNX27-Retromer or SNX17-Retriever were combined and analyzed with Metascape to represent DisGeneNET category enrichment, using a hypergeometric test and Benjamini-Hochberg correction, category enrichment p-value is represented in -Log10 scale.

Supplementary Figure 4

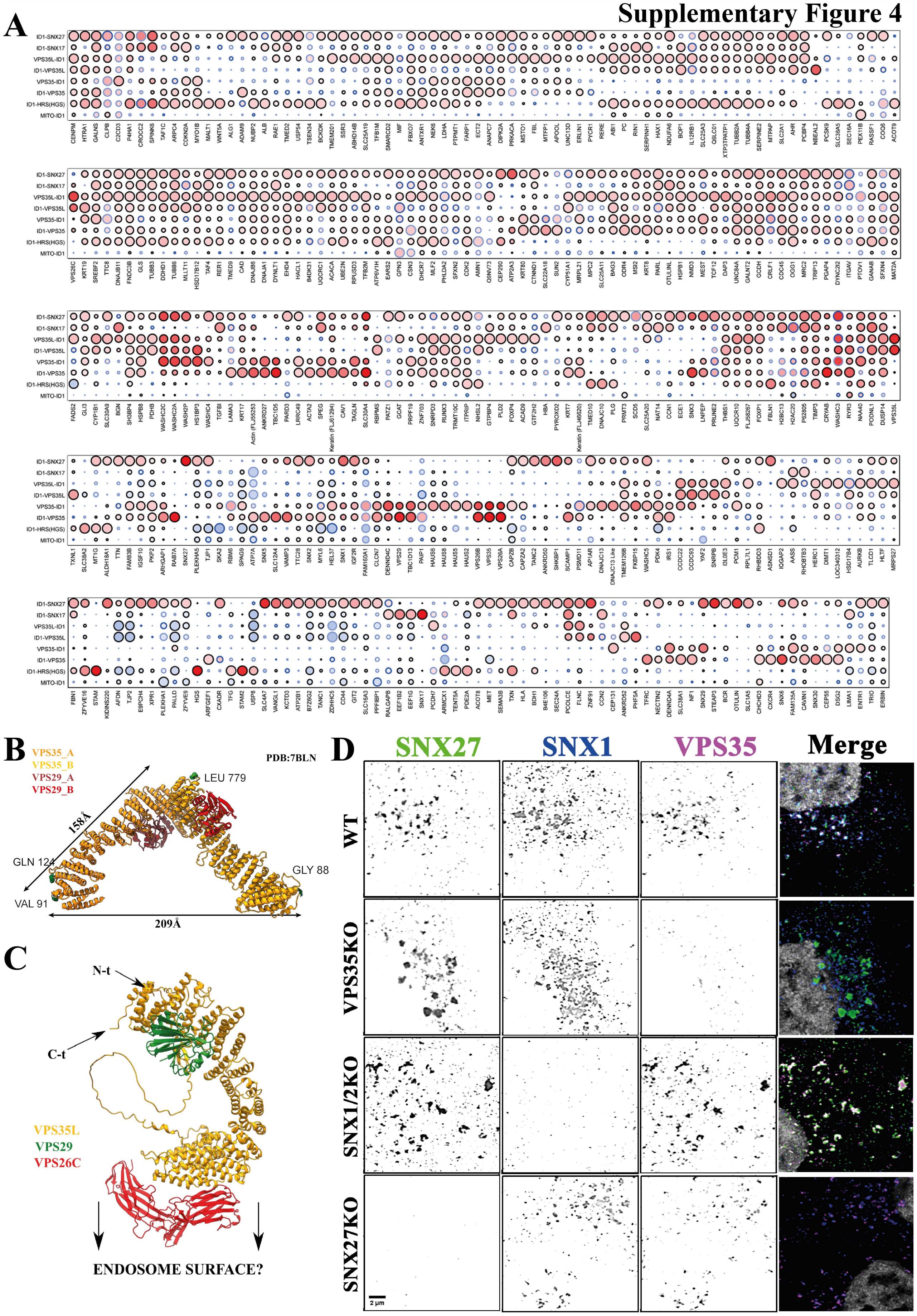

Supplementary Figure 4. Proteomic quantitative comparison among endosomal sub-domains; extended version. (A) Dot plot showing the comparison of FC (Log_2) and significance of detected proteins, along the different endosomal sub-domains as well Mito-BioID1. Only proteins with FC $\geq$ 0.8 and significant (p-value $\leq$ 0.05) in at least one proximity proteome were selected for the comparison. Filling colours represent the FC value, edge colours the p-value and the size symbolize the relative FC along the different sub-domains for that specific protein. **(B)** Representation of main length distances over the published structure of VPS35/VPS29 arch from the metazoan membrane-assembled Retromer:SNX3 complex modelled with human proteins (PDB:7BLN). **(C)** AlphaFold2 predictive model of Retriever complex. As Retriever complex binds the cargo adaptor SNX17 through its VPS26C subunit in the vicinity of the endosomal surface, the N and C termini of VPS35L might points far from the endosomal surface. This could explain why both Retriever BioID1 versions, ID1-VPS35L and VPS35L-ID1, strongly biotinylate WASH subunits and CCDC22/93, but not SNX17 cargo adaptor. **(D)** Images showing colocalization of Retromer (VPS35), SNX27 and SNX1 in HeLa parental, VPS35KO, SNX1/SNX2 double KO, and SNX27KO using endogenous antibodies and confocal imaging. Images were acquired on a Leica SP8 multi-laser point scanning confocal microscope with a 63x NA1.4 UV oil-immersion lens and using the Leica 'Lightning' mode for adaptive deconvolution to improve lateral resolution. Scale bar represents 2 μm.

Supplementary Figure 5

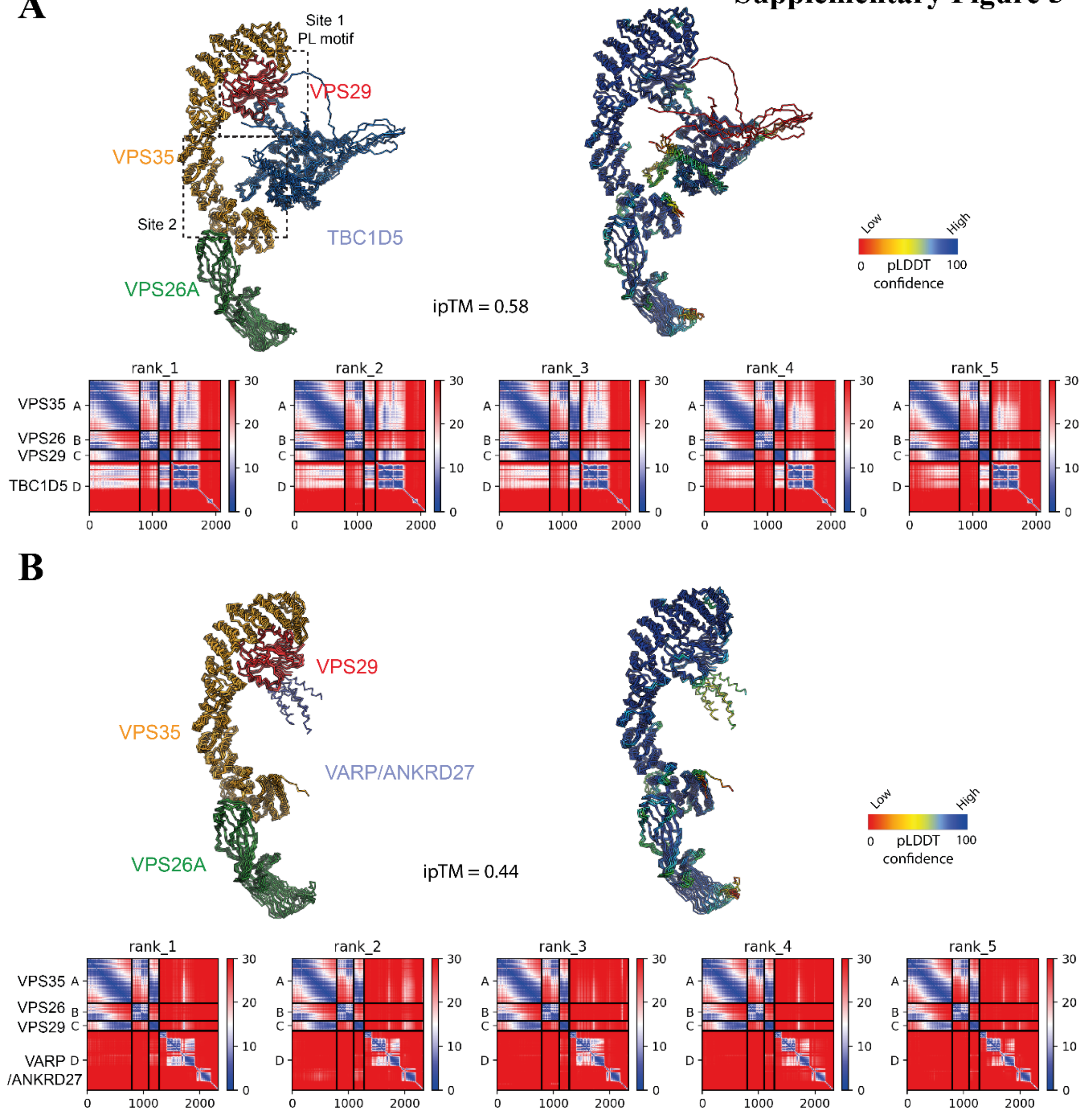

Supplementary Figure 5. Retromer interactions with TBC1D5 and VARP/ANKRD27. (A) AlphaFold2-predicted complex between human Retromer and TBC1D5 (Q92609). The top five ranked structures are aligned and shown in ribbon diagram. For clarity, the extended C-terminal disordered sequences of TBC1D5 are omitted from the images. The Left panel shows each chain coloured with VPS35 (orange), VPS26A (green), VPS29 (red) and TBC1D5 (blue). The right panel shows the structures coloured according to the pLDDT confidence score. Bottom panels show the predicted alignment error (PAE) plots for all five predictions of each complex. **(B)** AlphaFold2-predicted complex between human Retromer and VARP/ANKRD27 (Q96NW4). The top five ranked structures are aligned and shown in ribbon diagram. For clarity, only the primary binding regions of VARP are shown in the images. The Left panel shows each chain coloured with VPS35 (orange), VPS26A (green), VPS29 (red) and VARP (blue). The right panels show the structures coloured according to the pLDDT confidence score. Bottom panels show the PAE plots for all five predictions of each complex.

Supplementary Figure 6

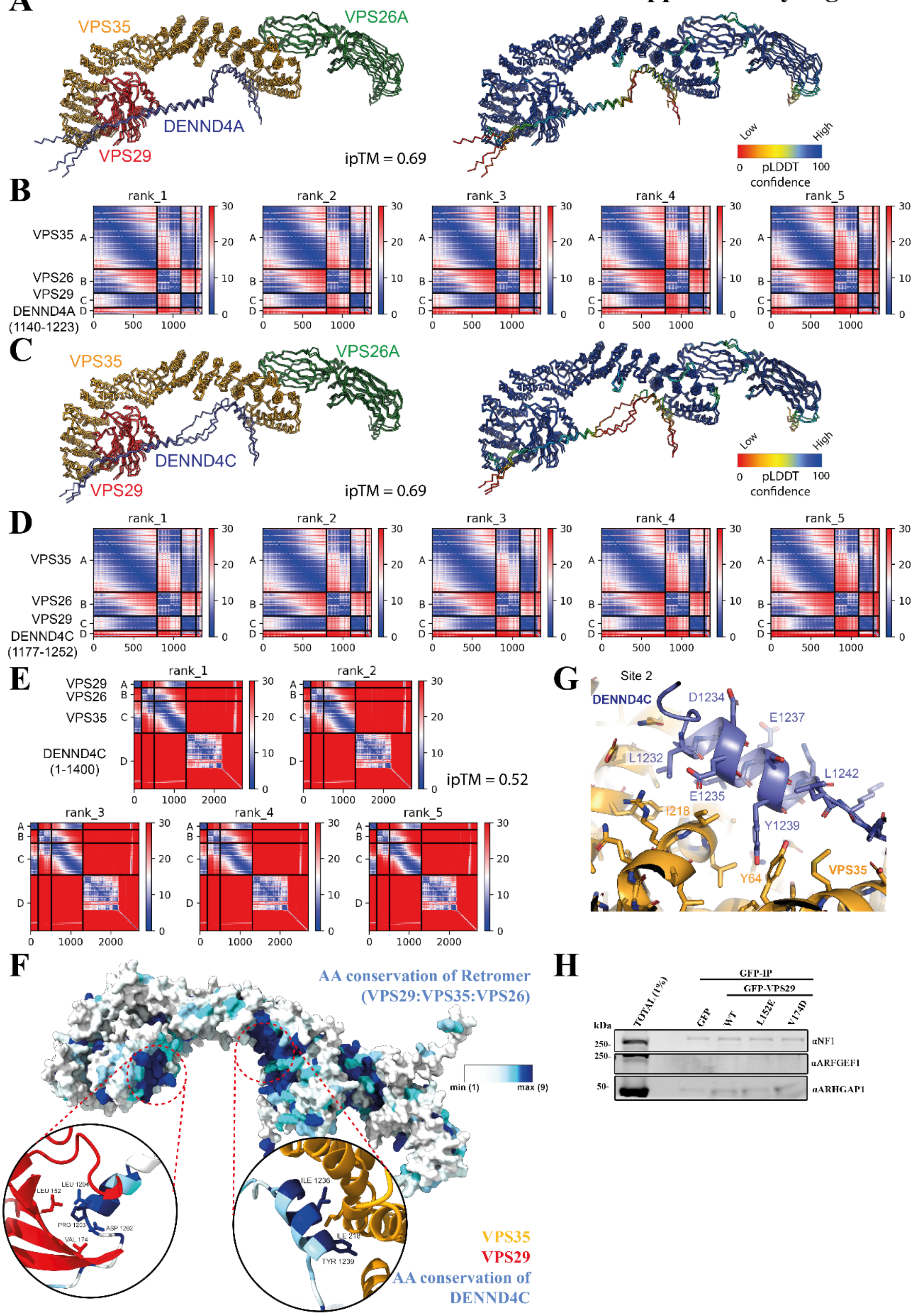

Supplementary Figure 6. Retromer interactions with DENND4A and DENND4C. (A-D) AlphaFold2-predicted complex between human Retromer and the binding regions of DENND4A residues 1140-1223 (Q7Z401) and DENND4C residues 1177-1252 (Q5VZ89). The top three ranked structures are aligned and shown in ribbon diagram. (A and C) The Left panel shows each chain coloured with VPS35 (orange), VPS26A (green), VPS29 (red) and DENND4A/C (blue). The right panels show the structures coloured according to the pLDDT confidence score. (B and D) Graphics show the predicted alignment error (PAE) plots for all five predictions of each complex. **(E)** Graphics show the PAE plots for all five predictions of each complex between Retromer and first 1400 amino acids from DENND4C (Q5VZ89) predicted by AlphaFold2. **(F)** Amino acid sequence conservation depicted over AlphaFold2 models of Retromer complex and DENND4C interacting domains calculated with ConSurf server applying default parameters. Main structure corresponds to Retromer trimer (VPS26:VPS29:VPS35) with the surface representation. Circular insets show magnifications for the 2 predicted binding sites between DENND4C and Retromer. Left inset shows the primary binding site consisting of a conserved PL motif (DENND4C ^{1203}PL^{1204}) interacting with the Leu152 containing hydrophobic cavity of VPS29. Right inset shows a second binding site predicted between a short DENND4C α-helical stretch (residues 1232-1243) with α-helices towards the amino-termini of VPS35. For clarity, ribbon representation was selected and only DENND4C sequence conservation at binding sites was shown. **(G)** Detailed view of Alphafold2 model of the secondary DENND4C binding site on the VPS35 subunit of Retromer. **(H)** GFP based co-immunoprecipitation (co-IP) of GFP-VPS29 wild-type (WT) and hydrophobic pocket mutants after transient transfection in HEK293T cells. Additional GTPase regulators found in the Retromer proximity proteomes (Figure 5A); neurofibromin (NF1), ARHGAP1 and ARFGEF1, contrary to DENND4A/C or TBC1D13 show weak interaction under GFP-VPS29 co-IP.

Supplementary Figure 7

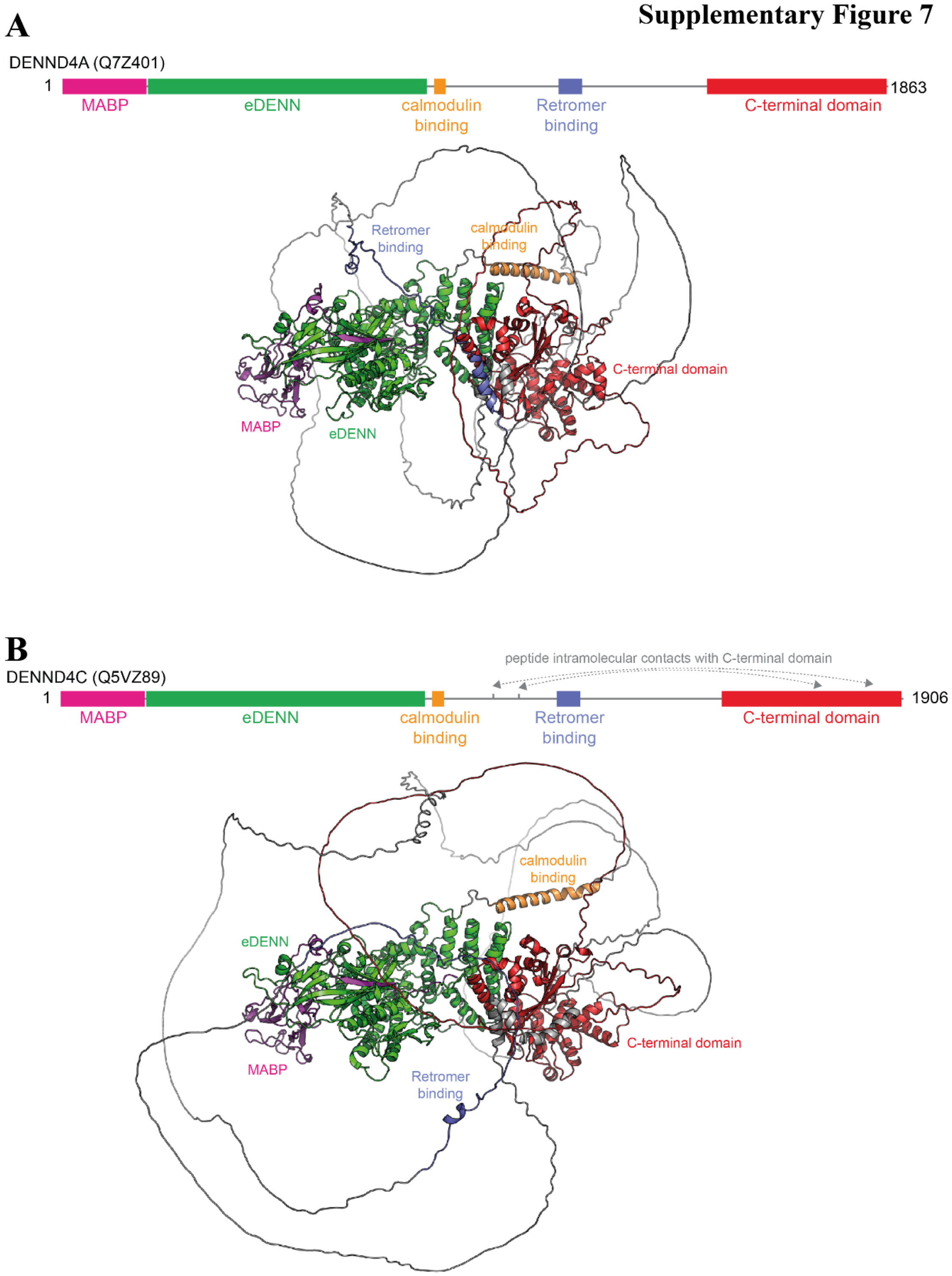

Supplementary Figure 7. AlphaFold2 model of full length human DENND4A and DENND4C. (A-B) Top diagrams show domain architecture of human DENND4A (Q7Z401) (A) or DENND4C (Q5VZ89) (B) based on AlphaFold2 predictions of tertiary structures. Bottom diagrams show tertiary structure of DENND4A/ and DENND4C derived from AlphaFold2 prediction. Specific domains and binding sequences are coloured as indicated. MABP domain (MVB12-associated β-prism domain); eDENN domain (extended DENN domain); C-terminal domain (a globular domain that is structurally unique to DENND4 homologues).

Supplementary Figure 8

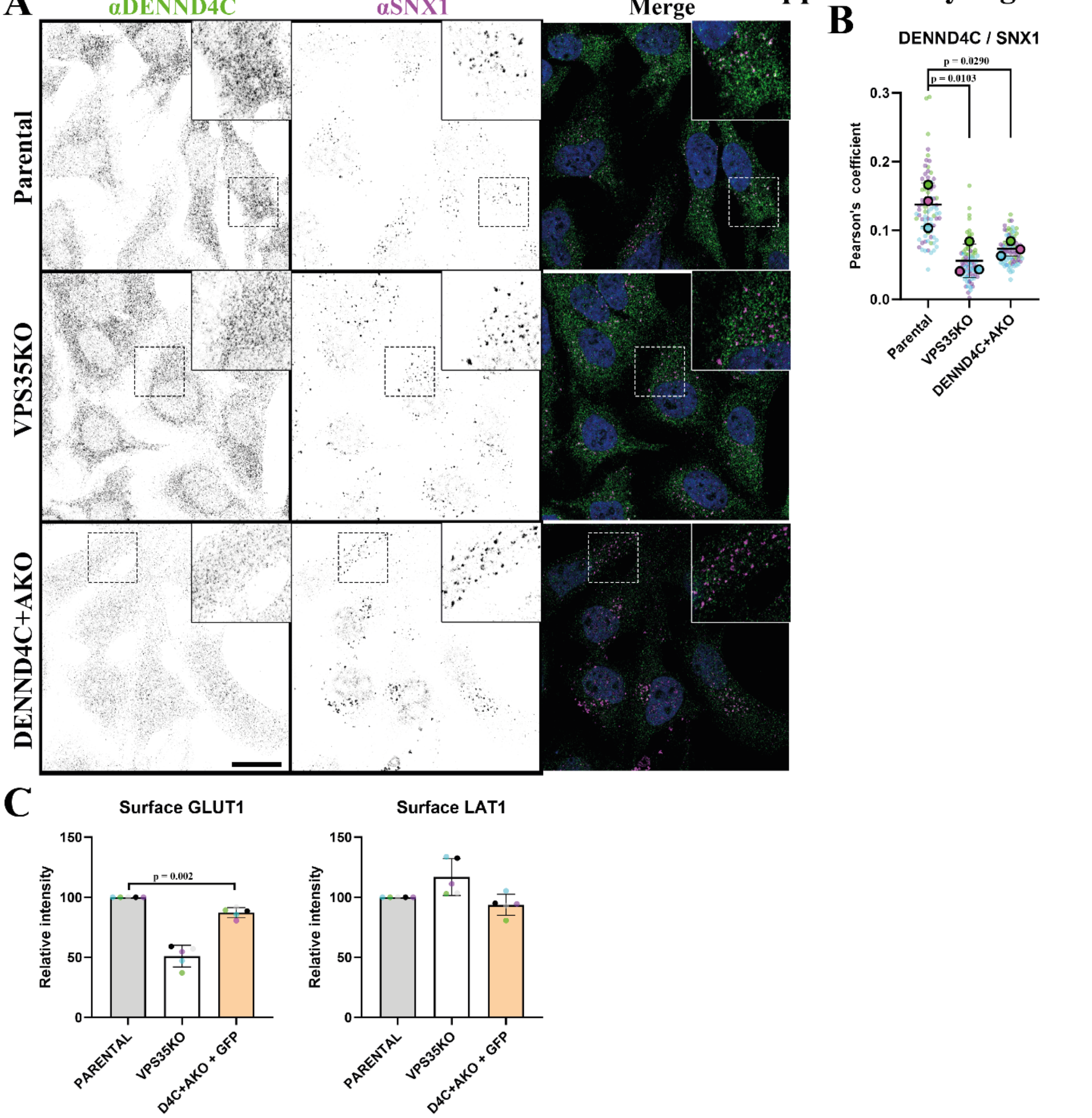

Supplementary Figure 8. Extended figure on role of DENND4A/C in Retromer's cell biology. (A) Confocal images showing colocalization of endogenous DENND4C and SNX1 in HeLa parental, VPS35KO and DENND4C and DENND4A double KO (DENND4C+A KO). Scale bar - 20 μm. **(B)** Quantitation and statistical analysis of colocalization between DENND4C and SNX1 using Pearson's coefficient, showing a significant reduction in both KO lines. n = 3 independent experiments. 1-way ANOVA with Dunnett's multiple comparison test, data presented as mean values relative to WT and error bars represent SD. Only changes with $p < 0.05$ are shown. **(C)** Quantitation and statistical analysis of GLUT1 and LAT1 surface levels in Hela parental versus DENND4C+A KO from Figure 5C. n = 5 independent experiments. Two-sided t-test analysis, data presented as mean values relative to WT and error bars represent SD. Data shows a moderate but significant reduction in GLUT1 surface levels. Only changes with $p < 0.05$ are shown. Values for VPS35KO are shown just for visual comparison.

Supplementary Figure 9

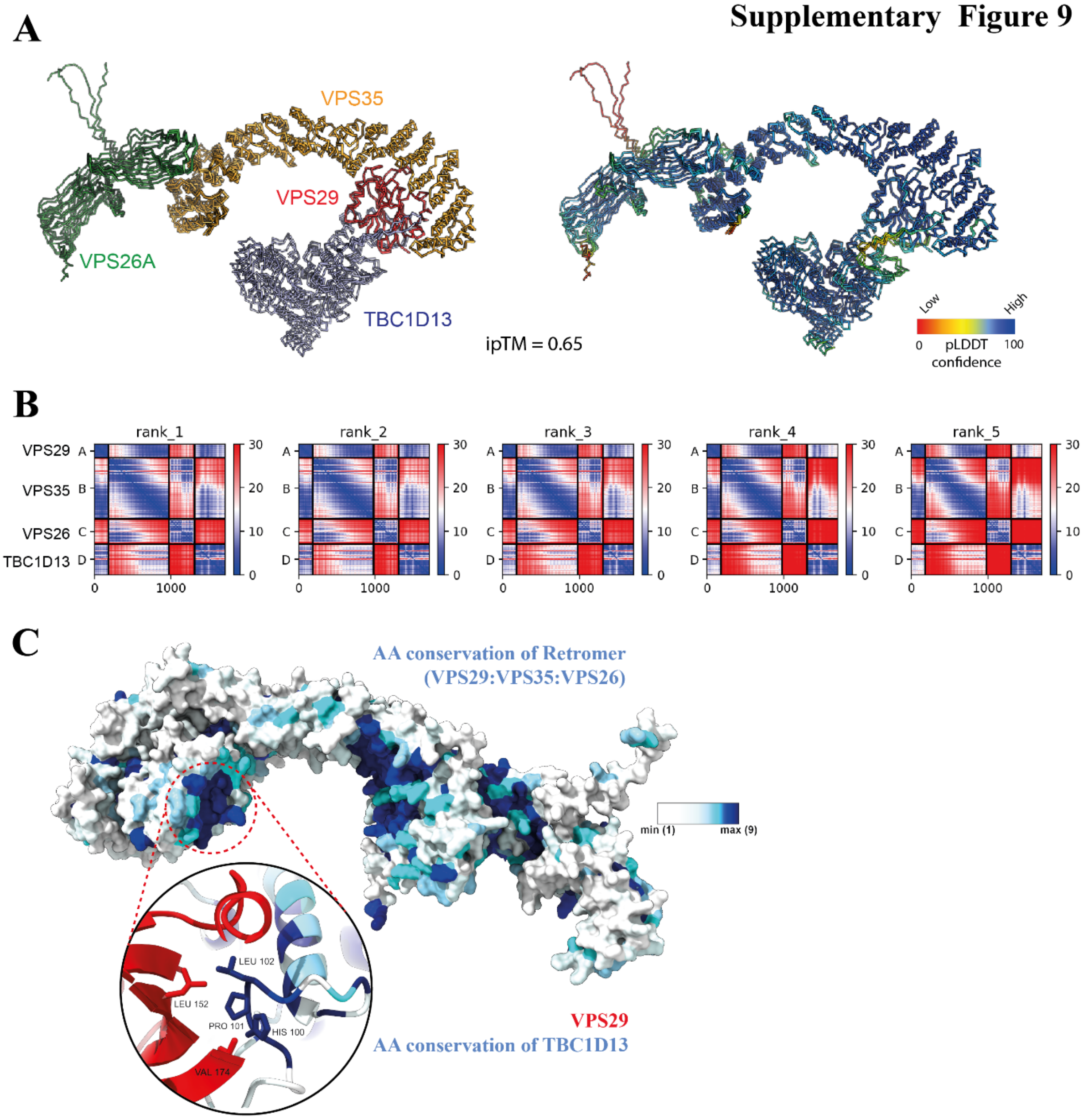

Supplementary Figure 9. Retromer interactions with TBC1D13. (A) AlphaFold2-predicted complex between human Retromer and TBC1D13 (Q9NVG8). The top three ranked structures are aligned and shown in ribbon diagram. The Left panel shows each chain coloured with VPS35 (orange), VPS26A (green), VPS29 (red) and TBC1D13 (blue). The right panel show the structures coloured according to the pLDDT confidence score. **(B)** Predicted alignment error (PAE) plots for all five predictions from (A). **(C)** Amino acid sequence conservation depicted over AlphaFold2 model of Retromer complex and TBC1D13 interacting domains calculated with ConSurf server applying default parameters. Main structure corresponds to Retromer trimer with the surface representation. Circular inset shows magnification for the main binding site consisting of a conserved PL motif (TBC1D13 ^{101}PL^{102}) interacting with the Leu152 containing hydrophobic cavity of VPS29. For clarity, ribbon representation was selected and only TBC1D13 sequence conservation at binding site was shown.

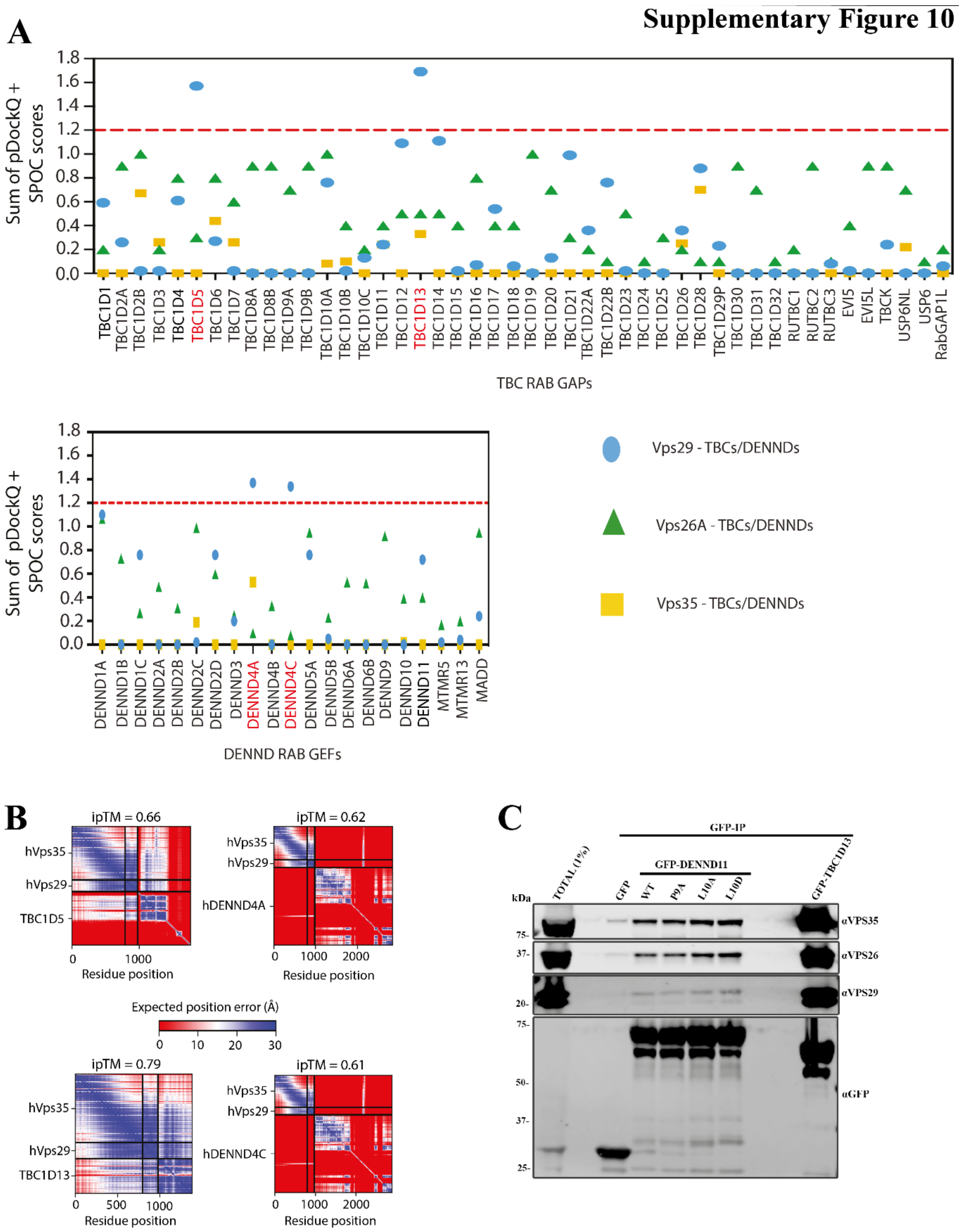

Supplementary Figure 10. Retromer as a hub for RAB GTPase switch regulation: Extended information on VPS29 interaction screen. (**A**) Sum of pDockQ plus SPOC scores for all TBC and DENND domain containing RAB GAPs and GEFs respectively against VPS29, VPS26A and VPS35 subunits of human Retromer. **(B)** Top ranked PAE plots for VPS35:VPS29 dimer for their AlphaFold2 predicted association with TBC1D5, DENND4A, TBC1D13 and DENND4C. **(C)** GFP based co-immunoprecipitation (co-IP) of GFP-DENND11 wild-type (WT) and PL motive mutants compared to GFP-TBC1D13 after transient transfection in HEK293T cells.

Supplementary Figure 11

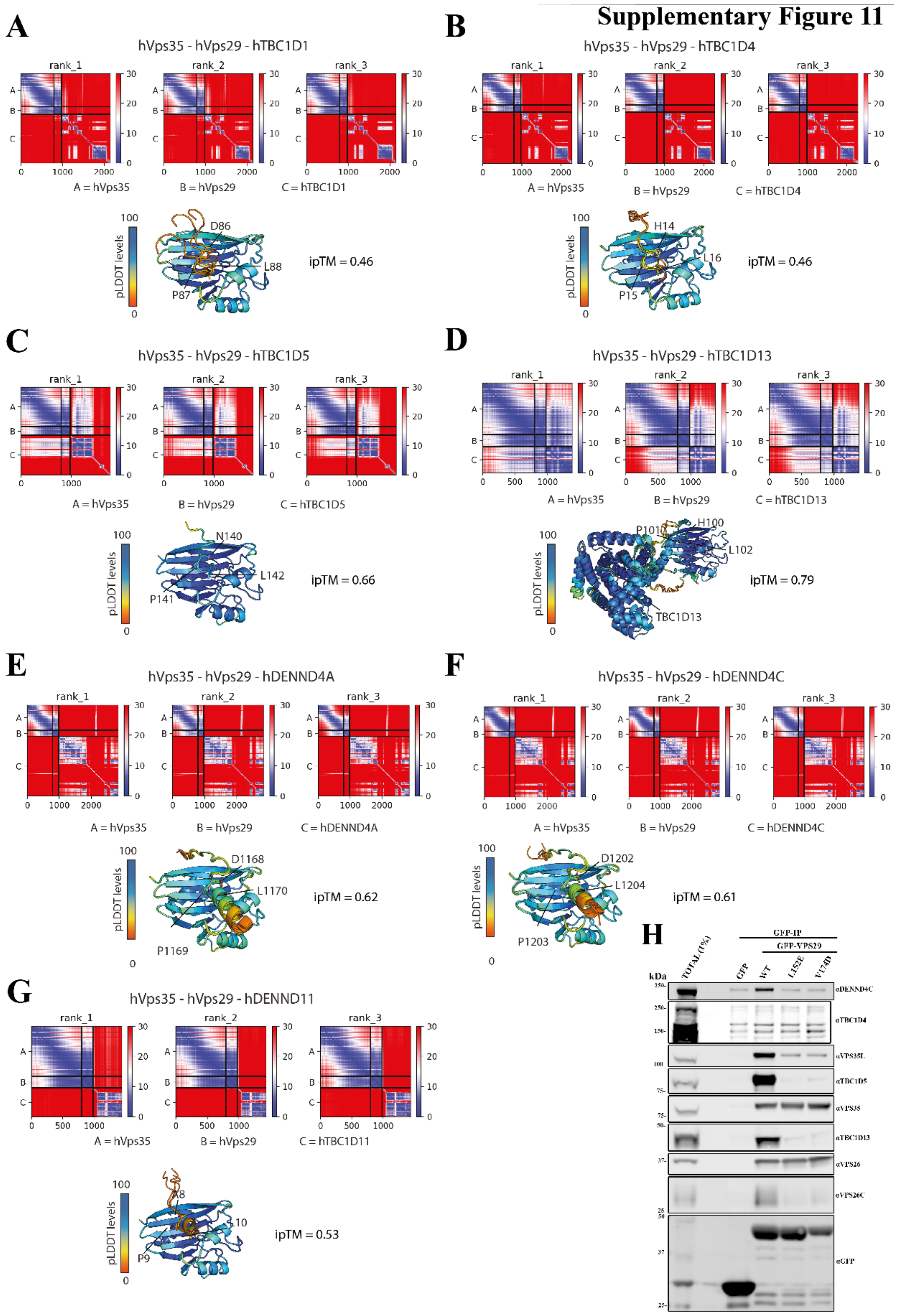

Supplementary Figure 11. TBC1D1/4 interaction with Retromer. (A-G) Predicted alignment error (PAE) plots and pLDDT structural representation for all seven AlphaFold2 predictions of VPS35:VPS29 association with (A) TBC1D1, (B) TBC1D4, (C) TBC1D5, (D) TBC1D13, € DENND4A, (F) DENND4C, and (G) DENND11. **(H)** GFP based co-immunoprecipitation (co-IP) of GFP-VPS29 wild-type (WT) and

hydrophobic pocket mutants after transient transfection in HEK293T cells. Here we blotted for TBC1D4, which contrary to DENND4A/C or TBC1D13 show weak interaction under GFP-VPS29 co-IP.

Supplementary Figure 12

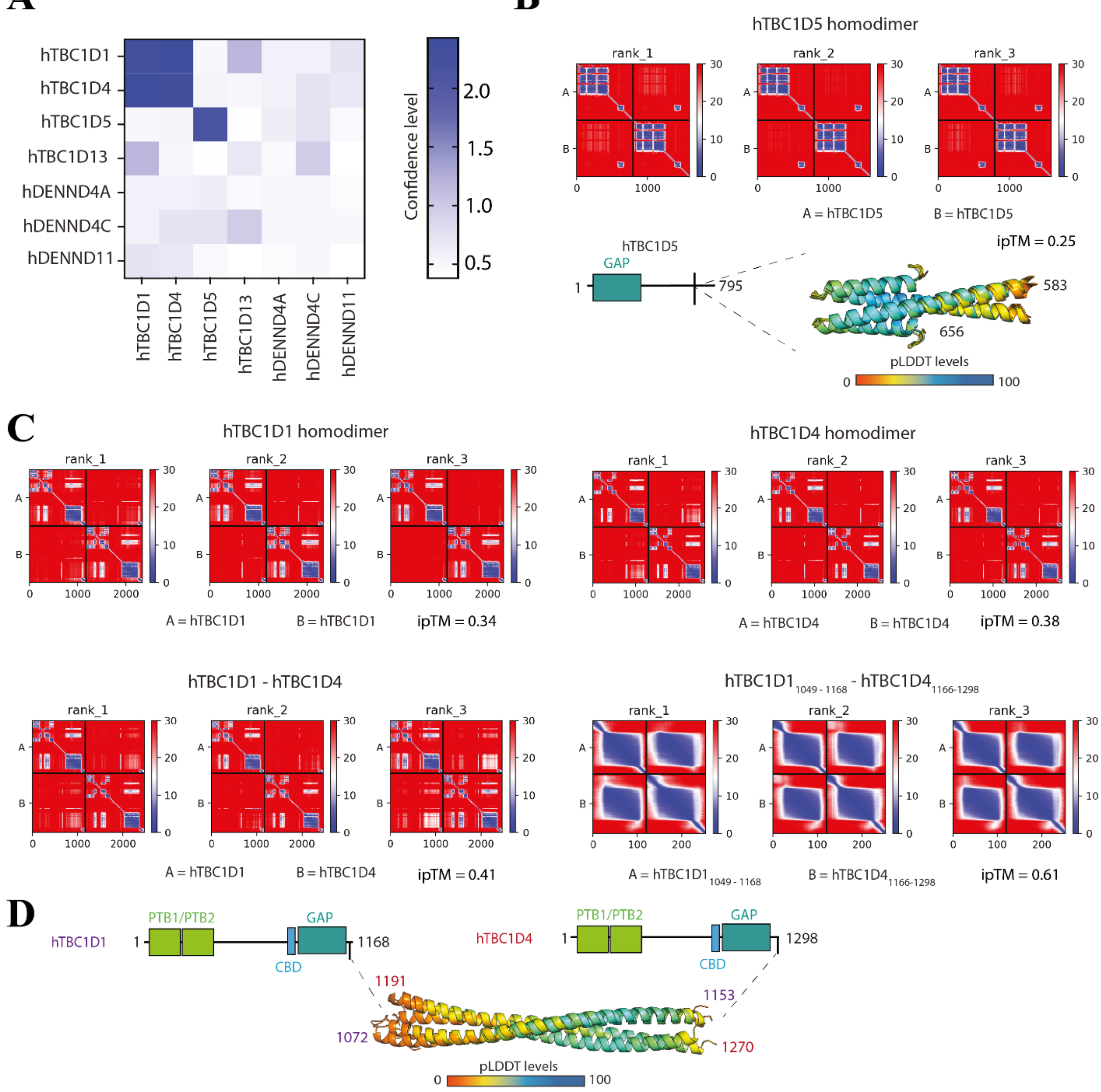

Supplementary Figure 12. Predicted mechanism for TBC1D1-TBC1D4 heterodimer and TBC1D5 homodimer formation. (A) Confidence level predictor of homo- and heterodimer formation with a network of Retromer binding TBC and DENND proteins. **(B)** First three ranked PAE plots of the predicted formation of a TBC1D5 homodimer through carboxy-terminal coiled-coil interactions. **(C)** First three ranked PAE plots of the predicted formation of TBC1D1 homodimer, TBC1D4 homodimer, full length TBC1D1 and TBC1D4 heterodimer, and heterodimer of the carboxy-terminal regions of TBC1D1 and TBC1D4. **(D)** pLDDT levels depicted on the predicted AlphaFold model of the coiled-coil carboxy-terminal TBC1D1 and TBC1D4 heterodimer.

Table S1: gRNAs

PRIMER NAME:	GENE:	EXON:	SEQUENCE (**sgRNA in bold**):
HRS.guide.Exon3.F	HRS/HGS	3	CACC**GGGCATACAAGGCGACGTGT**
HRS.guide.Exon3.R	HRS/HGS	3	AAACACACGTCGCCTTGTATGCCC
DENND4C.guide.Exon5.F	DENND4C	5	CACCG**CACTCAATAGTAGCTCCCAT**
DENND4C.guide.Exon5.R	DENND4C	5	AAACATGGGAGCTACTATTGAGTGC
DENND4A.guide.Exon4.F	DENND4A	4	CACCG**AAACATGACTCAGAATACGT**
DENND4A.guide.Exon4.R	DENND4A	4	AAACACGTATTCTGAGTCATGTTTC

Table S2: Reagent or Resource

**REAGENT or RESOURCE**	**SOURCE or REFERENCE**	**IDENTIFIER**
**Bacterial Strains**		
E. coli DH5α	Invitrogen	18265017
E. coli BL21(DE3)	Merck Australia	CMC0016
XL1-Blue	Agilent	200249
NEB® 5-alpha Competent E. coli (High Efficiency)	New England Biolabs	C2987H
**Chemicals**		
Magnesium sulfate anhydrous	Sigma Aldrich	208094
Magnesium chloride hexahydrate	ChemSupply Australia	MA029
Glycerol	ChemSupply Australia	GA010
Glucose	Thermo Fisher Scientific	AJA783
α-Lactose monohydrate	Sigma Aldrich	L3625
Ammonium sulfate	ChemSupply Australia	AA014

Potassium dihydrogen orthophosphate anhydrous	ChemSupply Australia	PA009
di-sodium hydrogen orthophosphate anhydrous	Merck Life Science	1065860500
Benzamidine hydrochloride hydrate	Sigma Aldrich	B6506
Deoxyribonuclease I (DNase I)	Sigma Aldrich	DN25
Talon® resin	Clontech	635503
Glutathione Sepharose 4B	GE Healthcare	GEHE17-0756-0
Polyethylene glycol 4000	Hampton Research	HR2-529
**Recombinant DNA**		
Plasmid: pGEX4P1 – Vps29	This study	N/A
Plasmid: pET28a – Vps35	This study	N/A
Plasmid: pEG28a – Vps26A	This study	N/A
Plasmid: pGEX6P1 – TBC1D13	This study	N/A
Plasmid: psPAX2 (lentiviral packing)	Cullen lab	N/A
Plasmid: pMD2.G (lentiviral envelope)	Cullen lab	N/A
Plasmid: pLVX-CMV-MCS-EGFP-IRES-Puro	Yamauchi Laboratory	N/A
Plasmid: pLVX-CMV-MCS-PGK-Puro (pLVX-Puro)	Cullen lab	N/A
Plasmid: pLVX-Puro_FLAG-BioID1	This study	N/A
Plasmid: pLVX-Puro_TOM70-RFP-FLAG-BioID1	This study	N/A
Plasmid: pLVX-Puro_FLAG-BioID1-linker-VPS35	This study	N/A
Plasmid: pLVX-Puro_VPS35-linker-FLAG-BioID1	This study	N/A
Plasmid: pLVX-Puro_FLAG-BioID1-linker-VPS35L	This study	N/A
Plasmid: pLVX-Puro_VPS35L-linker-FLAG-BioID1	This study	N/A
Plasmid: pLVX-Puro_FLAG-BioID1-linker-HRS	This study	N/A
Plasmid: pLVX-Puro_FLAG-BioID1-linker-SNX17	This study	N/A
Plasmid: pLVX-Puro_FLAG-BioID1-linker-SNX27	This study	N/A
Plasmid: pEGFP-C1	Cullen lab	N/A
Plasmid: pEGFP-C1_VPS29	Cullen lab	N/A
Plasmid: pEGFP-C1_VPS29 L152E mut	Cullen lab	N/A

Plasmid: pEGFP-C1_VPS29 V174D mut	Cullen lab	N/A
Plasmid: pEGFP-N1	Cullen lab	N/A
Plasmid: pEGFP-N1_VPS35	Cullen lab	N/A
Plasmid: pEGFP-N1_VPS35 I218D mut	This study	N/A
Plasmid: pEGFP-C1_DENND4C_Frag:1087-1271	Cullen lab	N/A
Plasmid: pEGFP-C1_DENND4C_Frag:1087-1271 L1204D mut	This study	N/A
Plasmid: pEGFP-C1_DENND4C_Frag:1087-1271 L1204A mut	This study	N/A
Plasmid: pEGFP-C1_DENND4C_Frag:1087-1271 Y1239D mut	This study	N/A
Plasmid: pEGFP-C1_DENND4C_Frag:1087-1271 Δ helix mut	This study	N/A
Plasmid: pLVX-Puro_GFP-Dennd4C (Mouse)	This study (original DNA Steinberg Lab)	N/A
Plasmid: pLVX-Puro_GFP-Dennd4C (Mouse) L1201A mut	This study	N/A
Plasmid: pEGFP-C1_TBC1D13	This study	N/A
Plasmid: pEGFP-C1_TBC1D13 P101A mut	This study	N/A
Plasmid: pEGFP-C1_TBC1D1	This study (original DNA Tavare Lab)	N/A
Plasmid: pEGFP-C1_TBC1D4	This study (original DNA Tavare Lab)	N/A
Plasmid: p3xFLAG_mCherry-TBC1D4	Tavare Lab	N/A
Plasmid: pEGFP-C1_TBC1D1 P87A mut	This study	N/A
Plasmid: p3xFLAG_mCherry-TBC1D4 P15A mut	This study	N/A
Plasmid: pSpCas9(BB)-2A-Puro (pX459)	Cullen lab	N/A
Plasmid: pEGFP-C1_DENND11	This study	N/A
Plasmid: pEGFP-C1_DENND11 P9A mut	This study	N/A
Plasmid: pEGFP-C1_DENND11 L10A mut	This study	N/A
Plasmid: pEGFP-C1_DENND11 L10D mut	This study	N/A
**Software**		
Phenix	The Phenix Industrial Consortium	https://phenix-online.org/
Pymol	Schrodinger, USA.	https://pymol.org/2/

Consurf	(1)	https://consurf.tau.ac.il/consurf_index.php
AlphaFold2 Multimer	(2)	https://github.com/deepmind/alphafold
ColabFold and ColabFold batch	(3)	https://github.com/sokrypton/ColabFold
ImageJ FIJI	NIH	https://imagej.net/
Volocity 6.3	PerkinElmer	N/A
Image Studio	LI-COR Biosciences	N/A
ChimeraX 1.8	NIH	https://www.cgl.ucsf.edu/chimerax/
**Other**		
HiLoad^{TM} Superdex200 16/600 PG	GE Healthcare	GEHE28-9893-35

Table S3. Reference table for AlphaFold models.

**Model**	**ModelArchive ID**	**DOI**
Vps35_Vps29_DENND4A	ma-gpsxe	10.5452/ma-gpsxe
Vps35_Vps29_DENND4C	ma-kixo0	10.5452/ma-kixo0
Vps35_Vps29_DENND11	ma-xv6fg	10.5452/ ma-xv6fg
Vps35_Vps29_TBC1D1	ma-mupfy	10.5452/ma-gpsxe
Vps35_Vps29_TBC1D4	ma-igov0	10.5452/ma-igov0
Vps35_Vps29_TBC1D5	ma-uf8h5	10.5452/ma-uf8h5
Vps35_Vps29_TBC1D13	ma-rkiek	10.5452/ma-rkiek
TBC1D1_homodimer	ma-ce92s	10.5452/ma-ce92s
TBC1D1_TBC1D4	ma-4yqbq	10.5452/ma-4yqbq
TBC1D1tail_TBC1D4tail	ma-xv4vj	10.5452/ma-xv4vj
TBC1D4_homodimer	ma-8wube	10.5452/ma-8wube
TBC1D5_homodimer	ma-qv4wj	10.5452/ma-qv4wj
Vps26A_Vps35_Vps29_DENND4C1to1400	ma-77fz1	10.5452/ma-77fz1
Vps26A_Vps35_Vps29_TBC1D5	ma-h364h	10.5452/ma-h364h
Vps29_Vps35_Vps26A_TBC1D13	ma-eazq0	10.5452/ma-eazq0
Vps35_Vps26A_Vps29_DENND4A1140to1223	ma-b9i08	10.5452/ma-b9i08
Vps35_Vps26A_Vps29_DENND4C1177to1252	ma-c4705	10.5452/ma-c4705
Vps35_Vps26A_Vps29_VARP	ma-0p47r	10.5452/ma-0p47r

REFERENCES:

1. Ashkenazy, H. *et al.* ConSurf 2016: an improved methodology to estimate and visualize evolutionary conservation in macromolecules. *Nucleic Acids Res* **44**, W344–W350 (2016).
2. Evans, R. *et al.* Protein complex prediction with AlphaFold-Multimer. *bioRxiv* 2021.10.04.463034 (2022) doi:10.1101/2021.10.04.463034.
3. Mirdita M, Ovchinnikov S, Steinegger M. (2022). ColabFold – Making protein folding accessible to all. Nat Methods 19, 679-682.
