# Supplementary material for: Mapping of endosomal proximity proteomes reveals Retromer as a hub for RAB GTPase regulation
Source: Nat Commun. 2025 Jul 30;16:6990. doi: 10.1038/s41467-025-61802-1 (PMC12311110; doi:10.1038/s41467-025-61802-1)
Supplement: Supplementary file 2 — Description of Additional Supplementary Files [file 41467_2025_61802_MOESM2_ESM.pdf]

Description of Additional Supplementary Files

Supplementary Data 1: Excel file with the Proximity Proteomic Data.
Raw Proximity Proteomics Data for all the BioID1 conditions. Output data is displayed for each protein across all the conditions obtained in 6 independent experiments, including gene ontology classifications, peptide counts, protein coverage, Log2 fold change, two-sided unpaired t-test p-values and false discovery rate (FDR). This dataset was used to generate the plots in Figures 1-3.
