# Supplementary material for: Mapping of endosomal proximity proteomes reveals Retromer as a hub for RAB GTPase regulation
Source: Nat Commun. 2025 Jul 30;16:6990. doi: 10.1038/s41467-025-61802-1 (PMC12311110; doi:10.1038/s41467-025-61802-1)
Supplement: Supplementary file 4 — Reporting Summary [file 41467_2025_61802_MOESM4_ESM.pdf]

nature portfolio

Corresponding author(s): Carlos Antón-Plágaro, Peter J. Cullen

Last updated by author(s): Jun 11, 2025

Reporting Summary

Nature Portfolio wishes to improve the reproducibility of the work that we publish. This form provides structure for consistency and transparency in reporting. For further information on Nature Portfolio policies, see our Editorial Policies and the Editorial Policy Checklist.

Statistics

For all statistical analyses, confirm that the following items are present in the figure legend, table legend, main text, or Methods section.

n/a	Confirmed	
☐	☒	The exact sample size (*n*) for each experimental group/condition, given as a discrete number and unit of measurement
☐	☒	A statement on whether measurements were taken from distinct samples or whether the same sample was measured repeatedly
☐	☒	The statistical test(s) used AND whether they are one- or two-sided<br>*Only common tests should be described solely by name; describe more complex techniques in the Methods section.*
☒	☐	A description of all covariates tested
☐	☒	A description of any assumptions or corrections, such as tests of normality and adjustment for multiple comparisons
☐	☒	A full description of the statistical parameters including central tendency (e.g. means) or other basic estimates (e.g. regression coefficient) AND variation (e.g. standard deviation) or associated estimates of uncertainty (e.g. confidence intervals)
☐	☒	For null hypothesis testing, the test statistic (e.g. *F*, *t*, *r*) with confidence intervals, effect sizes, degrees of freedom and *P* value noted<br>*Give P values as exact values whenever suitable.*
☒	☐	For Bayesian analysis, information on the choice of priors and Markov chain Monte Carlo settings
☒	☐	For hierarchical and complex designs, identification of the appropriate level for tests and full reporting of outcomes
☐	☒	Estimates of effect sizes (e.g. Cohen's *d*, Pearson's *r*), indicating how they were calculated

Our web collection on statistics for biologists contains articles on many of the points above.

Software and code

Policy information about availability of computer code

Data collection	Confocal microscopy: LAS AF and LAS X (Leica Microsystems).<br>Spining disk high resolution microscopy: cellSens Dimension (Olympus).<br>Western blot scanning: LI-COR Image Studio (LI-COR Biosciences).<br>Isothermal titration calorimetry was determined using a Microcal PEAQ-ITC (Malvern).<br>X-ray diffraction data were carried out at the Australian Synchrotron MX2 beamline at 100 K.
Data analysis	Immunofluorescence microscopy image preparation and analysis: Volocity version 6.3.1 and ImageJ (FiJI) 2.9.0.<br>Western Blot analysis: LI-COR Image Studio.<br>Statistical analysis and graph preparation: GraphPad Prism 10.<br><br>For the proteomic analysis the raw data files were processed and quantified using Proteome Discoverer software v2.1 (Thermo Scientific) with the SEQUEST HT algorithm and searching against the UniProt Human database (downloaded 2021-01-14; 178486 sequences). For statistical analysis, standard t-test were used. Volcano plots were generated with VolcanoseR2 webapp or GraphPad Prism 9 software (LaJolla, CA). Gene ontology analysis of the proteins identified was performed using Metascape webapp and Cytoscape 3.9 software to represent pathway enrichment, DisGeneNET category enrichment, and protein-protein interaction (PPI) networks. Dot plot diagrams were generated using the platform prohits-viz.org.<br><br>Protein structural models were generated with AphaFold2 ColabFold or AphaFold3, and selected models were presented using PyMOL or ChimeraX 1.8.

X-ray diffraction data were indexed and integrated by AutoXDS and scaled using Aimless. Phase was solved by molecular replacement using Phaser. Refinements were performed using Phenix (The Phenix Industrial Consortium). Molprobity was used to evaluate the quality geometry of the refined and molecular figures were generated using PyMOL.

For manuscripts utilizing custom algorithms or software that are central to the research but not yet described in published literature, software must be made available to editors and reviewers. We strongly encourage code deposition in a community repository (e.g. GitHub). See the Nature Portfolio guidelines for submitting code & software for further information.

Data

Policy information about availability of data

All manuscripts must include a data availability statement. This statement should provide the following information, where applicable:

- Accession codes, unique identifiers, or web links for publicly available datasets
- A description of any restrictions on data availability
- For clinical datasets or third party data, please ensure that the statement adheres to our policy

AlphaFold modelling will be available in ModelArchive (www.modelarchive.org) after manuscript publication with the accession codes included in Table S3.

X-ray structure of VPS29 – DENND4C (amino acids 1189-1206) has been deposited at PDB with identification code 8VOD.

Proteomic data sets are included in an Excel spreadsheet as Supplementary Data and the raw data was uploaded at ProteomeXchange repository with identifier PXD061918.

All unprocessed immunoblots are provided in Source Data file.

All other data supporting the findings of this study are available from the corresponding authors on reasonable request.

Research involving human participants, their data, or biological material

Policy information about studies with human participants or human data. See also policy information about sex, gender (identity/presentation), and sexual orientation and race, ethnicity and racism.

Reporting on sex and gender	*Use the terms sex (biological attribute) and gender (shaped by social and cultural circumstances) carefully in order to avoid confusing both terms. Indicate if findings apply to only one sex or gender; describe whether sex and gender were considered in study design; whether sex and/or gender was determined based on self-reporting or assigned and methods used. Provide in the source data disaggregated sex and gender data, where this information has been collected, and if consent has been obtained for sharing of individual-level data; provide overall numbers in this Reporting Summary. Please state if this information has not been collected. Report sex- and gender-based analyses where performed, justify reasons for lack of sex- and gender-based analysis.*
Reporting on race, ethnicity, or other socially relevant groupings	*Please specify the socially constructed or socially relevant categorization variable(s) used in your manuscript and explain why they were used. Please note that such variables should not be used as proxies for other socially constructed/relevant variables (for example, race or ethnicity should not be used as a proxy for socioeconomic status). Provide clear definitions of the relevant terms used, how they were provided (by the participants/respondents, the researchers, or third parties), and the method(s) used to classify people into the different categories (e.g. self-report, census or administrative data, social media data, etc.) Please provide details about how you controlled for confounding variables in your analyses.*
Population characteristics	*Describe the covariate-relevant population characteristics of the human research participants (e.g. age, genotypic information, past and current diagnosis and treatment categories). If you filled out the behavioural & social sciences study design questions and have nothing to add here, write "See above."*
Recruitment	*Describe how participants were recruited. Outline any potential self-selection bias or other biases that may be present and how these are likely to impact results.*
Ethics oversight	*Identify the organization(s) that approved the study protocol.*

Note that full information on the approval of the study protocol must also be provided in the manuscript.

Field-specific reporting

Please select the one below that is the best fit for your research. If you are not sure, read the appropriate sections before making your selection.

☒ Life sciences ☐ Behavioural & social sciences ☐ Ecological, evolutionary & environmental sciences

For a reference copy of the document with all sections, see nature.com/documents/nr-reporting-summary-flat.pdf

Life sciences study design

All studies must disclose on these points even when the disclosure is negative.

Sample size	Sample size calculations were not performed. All quantified experiments were performed using at least 3 independent biological experiments.
Data exclusions	No data was excluded from this study.
Replication	All quantified experiments were performed using at least 3 independent biological repeats. The reproducibility of findings is displayed by the individual datapoints on graphs.
Randomization	Samples were allocated into experimental groups based on their genotype (parental/wild-type, DENND4C+A KO and rescue).
Blinding	Blinding was not used in this study

Reporting for specific materials, systems and methods

We require information from authors about some types of materials, experimental systems and methods used in many studies. Here, indicate whether each material, system or method listed is relevant to your study. If you are not sure if a list item applies to your research, read the appropriate section before selecting a response.

Materials & experimental systems

n/a	Involved in the study	
☐	☒	Antibodies
☐	☒	Eukaryotic cell lines
☒	☐	Palaeontology and archaeology
☒	☐	Animals and other organisms
☒	☐	Clinical data
☒	☐	Dual use research of concern
☒	☐	Plants

Methods

n/a	Involved in the study	
☒	☐	ChIP-seq
☒	☐	Flow cytometry
☒	☐	MRI-based neuroimaging

Antibodies

Antibodies used

Primary antibodies against the following targets were used: β-Actin (Sigma-Aldrich, A1978; 1:5000 WB, 1:1000 IF), GAPDH (Sigma-Aldrich, G9545; 1:5000 WB), GAPDH (Sigma-Aldrich, G8795; 1:5000 WB), EEA1 (Cell Signalling; 3288S; 1:200 IF), GFP (Roche; 11814460001; clones 7.1/13.1; 1:1000 WB), mCherry/RFP (Abcam, 167453; 1:1000 WB), FLAG-M2 (Sigma, F1804; 1:1000 WB, 1:200 IF), GLUT1 (Abcam; ab115730; 1:1000 WB; 1:400 IF), LAMP1 (Developmental Studies Hybridoma Bank; AB_2296838; clone H4A3; 1:400 IF), VPS29 (Santa Cruz; D-1; sc-398874; 1:500 WB), VPS35 (Abcam, ab97545; 1:1000 WB, 1:400 IF), VPS35 (Antibodies.com, A83699; 1:400 IF), VPS26 (Abcam, ab23892; 1:1000 WB), VPS35L (Abcam, ab97889; 1:1000 WB), VPS26C (Sigma-Aldrich, ABN87; 1:1000 WB), VPS35L (Invitrogen, PA5-28553; 1:200 IF), SNX1 (BD Transduction Lab, 611482; 1:1000 WB, 1:200 IF), SNX1 (Proteintech, 10304-1-AP; 1:200 IF), SNX2 (BD Transduction Lab ,611308; 1:1000 WB), SNX5 (Abcam, ab180520; 1:1000 WB), SNX6 (Santa Cruz, sc-365965; 1:1000 WB), SNX17 (Proteintech, 10275-1-AP; 1:1000 WB), SNX17 (Sigma, HPA043867, 1:100 IF), SNX27 (Proteintech, 16329-1-AP; 1:1000 WB), SNX27 (Abcam, ab77799; 1:100 IF), HRS/HGS (Enzo Life Sciences, ALX-804-382-C050; 1:2000 WB, 1:100 IF), STAM (Proteintech, 12434-1-AP; 1:2000 WB, 1:100 IF), FAM21 (Gift from Dan Billadeau; 1:2000 WB), Integrin-α5 (Abcam, ab150361; 1:1000 WB, 1;200 IF), DENND4C (Sigma-Aldrich, HPA014917, 1:1000 WB, 1:500 IF), DENND4C (StressMarq Bioscience, SMC-610, 1:100 IF), DENND4A (Abcam, ab117758, 1:500 WB), HAUS2 (ThermoFisher, PA5-31258; 1:500 WB), HAUS6 (Proteintech, 16933-1-AP; 1:1000 WB), HAUS8 (Abcam, ab95970; 1:250 WB), CAV1 (Proteintech, 16447-1-AP; 1:1000 WB), CAVIN1 (Proteintech, 18892-1-AP; 1:1000 WB), TBC1D13 (ThermoFisher, PA5-61110; 1:2000 WB, 1:200 IF), ARHGAP1 (Proteintech, 11169-1-AP; 1:1000 WB, 1:100 IF), VAMP3 (Proteintech, 10702-1-AP; 1:1000 WB, 1:200 IF), NF1 (Proteintech, 27249-1-AP, 1:1000 WB), ARFGEF1 (Abcam, A44423,1:1000 WB), TBC1D4 (Proteintech, 68063), KIDINS220 (Proteintech, 21856-1-AP, 1:1000 WB), ATP7A (Santa Cruz, sc-376467, 1:1000 WB), CTR1 (Abcam, ab129067, 1:1000 WB), LAT1 (Cell Signaling, 5347s, 1:1000 WB), TBC1D5 (Abcam, 203896, 1:1000 WB), VARP/ANKRD27 (Proteintech, 24034-1-AP, 1:1000 WB), RAB10 (Abcam, ab237703, 1:1000 WB, 1:200 IF).
Secondary antibodies for WB: 680nm and 800nm donkey anti-mouse and anti-rabbit fluorescent secondary antibodies (Invitrogen, A-21057, A3275; 1:20,000).
Secondary antibodies for IF: 405nm, 488nm, 568nm and 647nm AlexaFluor-labelled anti-mouse, anti-rabbit or anti-goat (Invitrogen; 1:400).

Validation

Validation was provided through the manufacturer's data sheet or through the published use and validation by our and other research groups.

Eukaryotic cell lines

Policy information about cell lines and Sex and Gender in Research

Cell line source(s)	HeLa and HEK293T cells were originally sourced from ATCC.

Authentication	Authentication for HEK293T was provided by ATCC, and HeLa was additionally authenticated by Eurofins Genomics Europe.
Mycoplasma contamination	HEK293T cell line and parental HeLa cells used for generation of all CRISPR edit cell lines were mycoplasma tested, which revealed no contamination.
Commonly misidentified lines (See ICLAC register)	This study did not use any commonly misidentified cell lines.

Plants

Seed stocks	*Report on the source of all seed stocks or other plant material used. If applicable, state the seed stock centre and catalogue number. If plant specimens were collected from the field, describe the collection location, date and sampling procedures.*
Novel plant genotypes	*Describe the methods by which all novel plant genotypes were produced. This includes those generated by transgenic approaches, gene editing, chemical/radiation-based mutagenesis and hybridization. For transgenic lines, describe the transformation method, the number of independent lines analyzed and the generation upon which experiments were performed. For gene-edited lines, describe the editor used, the endogenous sequence targeted for editing, the targeting guide RNA sequence (if applicable) and how the editor was applied.*
Authentication	*Describe any authentication procedures for each seed stock used or novel genotype generated. Describe any experiments used to assess the effect of a mutation and, where applicable, how potential secondary effects (e.g. second site T-DNA insertions, mosiacism, off-target gene editing) were examined.*
